# The comorbidity of depression and neurocognitive disorder in persons with HIV infection: call for investigation and treatment

**DOI:** 10.3389/fncel.2023.1130938

**Published:** 2023-04-28

**Authors:** Karl Goodkin, Teresa H. Evering, Albert M. Anderson, Ann Ragin, Cynthia L. Monaco, Christina Gavegnano, Ryan J. Avery, Sean B. Rourke, Lucette A. Cysique, Bruce J. Brew

**Affiliations:** ^1^Department of Psychiatry, School of Medicine, The University of Texas Rio Grande Valley, Harlingen, TX, United States; ^2^Institute of Neuroscience, School of Medicine, The University of Texas Rio Grande Valley, Harlingen, TX, United States; ^3^Division of Infectious Diseases, Department of Medicine, Weill Cornell Medicine, New York, NY, United States; ^4^Division of Infectious Diseases, Department of Medicine, School of Medicine, Emory University, Atlanta, GA, United States; ^5^Department of Radiology, Feinberg School of Medicine, Northwestern University, Chicago, IL, United States; ^6^Division of Infectious Diseases, Department of Medicine, University of Rochester School of Medicine and Dentistry, Rochester, NY, United States; ^7^Department of Microbiology and Immunology, University of Rochester School of Medicine and Dentistry, Rochester, NY, United States; ^8^Del Monte Institute of Neuroscience, University of Rochester School of Medicine and Dentistry, Rochester, NY, United States; ^9^Department of Pathology, Emory School of Medicine, Emory University, Atlanta, GA, United States; ^10^Department of Pharmacology, Emory School of Medicine, Emory University, Atlanta, GA, United States; ^11^Department of Chemical Biology, Emory School of Medicine, Emory University, Atlanta, GA, United States; ^12^Center for the Study of Human Health, Emory College of Arts and Sciences, Emory University, Atlanta, GA, United States; ^13^Atlanta Veteran’s Affairs Medical Center, Atlanta, GA, United States; ^14^Center for Bioethics, Harvard Medical School, Harvard University, Boston, MA, United States; ^15^Division of Nuclear Medicine, Department of Radiology, Feinberg School of Medicine, Northwestern University, Chicago, IL, United States; ^16^MAP Centre for Urban Health Solutions, Li Ka Shing Knowledge Institute, St. Michael’s Hospital, Toronto, ON, Canada; ^17^Department of Psychiatry, University of Toronto, Toronto, ON, Canada; ^18^School of Psychology, Faculty of Science, University of New South Wales, Sydney, NSW, Australia; ^19^Department of Neurology, Faculty of Medicine, University of New South Wales, Sydney, NSW, Australia; ^20^Department of Neurology, Faculty of Medicine, University of Notre Dame, Sydney, NSW, Australia

**Keywords:** HIV, depression, neurocognitive impairment, dopamine, inflammation, microbiome, neuroimaging

## Abstract

Depression and neurocognitive disorder continue to be the major neuropsychiatric disorders affecting persons with HIV (PWH). The prevalence of major depressive disorder is two to fourfold higher among PWH than the general population (∼6.7%). Prevalence estimates of neurocognitive disorder among PWH range from 25 to over 47% – depending upon the definition used (which is currently evolving), the size of the test battery employed, and the demographic and HIV disease characteristics of the participants included, such as age range and sex distribution. Both major depressive disorder and neurocognitive disorder also result in substantial morbidity and premature mortality. However, though anticipated to be relatively common, the comorbidity of these two disorders in PWH has not been formally studied. This is partly due to the clinical overlap of the neurocognitive symptoms of these two disorders. Both also share neurobehavioral aspects — particularly apathy — as well as an increased risk for non-adherence to antiretroviral therapy. Shared pathophysiological mechanisms potentially explain these intersecting phenotypes, including neuroinflammatory, vascular, and microbiomic, as well as neuroendocrine/neurotransmitter dynamic mechanisms. Treatment of either disorder affects the other with respect to symptom reduction as well as medication toxicity. We present a unified model for the comorbidity based upon deficits in dopaminergic transmission that occur in both major depressive disorder and HIV-associated neurocognitive disorder. Specific treatments for the comorbidity that decrease neuroinflammation and/or restore associated deficits in dopaminergic transmission may be indicated and merit study.

## Introduction

With regard to the general background to this article, major depressive disorder (MDD) has been noted to be the second most common cause of years lost to disability in the United States. Among persons with HIV (PWH), depressive disorders generally have been linked to decreased antiretroviral medication (ART) adherence and to worse clinical health outcomes. These outcomes also have been shown with HIV–associated neurocognitive disorder (HAND). Although standard antidepressant therapies for MDD are used to treat PWH, MDD in this population is generally under-diagnosed and under-treated. With respect to HAND, though HIV-associated dementia is rare in the current era of ART, mild neurocognitive disorder occurs with a prevalence rate of 12 to 25% and is defined by lesser but clinically significant functional impairment. Asymptomatic neurocognitive impairment without functional impairment occurs yet more frequently.

In clinical practice, it has become readily apparent that MDD co-occurs with HAND. However, current criteria for the diagnosis of HAND exclude or defer a diagnosis of MDD as a contributing factor or a confounding comorbidity, based upon the fact that cognitive dysfunction is one of the defining criteria of MDD. Thus, the actual prevalence of the comorbidity has not been formally studied, though it is expected to be relatively frequent. In addition, apathy had been noted as a defining characteristic of HAND (AAN, 1991), though it is less focused upon today. Apathy also relates to decreased interests in pleasurable activities, which is a defining characteristic of MDD. Hence, apathy is a common characteristic to both MDD and HAND. MDD is linked to the fronto-striatal and limbic brain injury known to occur with HAND. In addition, apathy specifically has been associated with underlying deficits in dopamine/dopaminergic (DA) transmission common to both MDD and HAND.

Cognitive dysfunction represents a core diagnostic and symptomatic criterion of MDD and is a principal determinant for lack of -recovery of function in daily life activities to baseline status. However, it is not required for the MDD diagnosis. Cognitive impairment increases with the severity of MDD, can occur in mild MDD, and is associated with a greater number and duration of prior depressive episodes. Moreover, MDD has been associated with the occurrence of dementia outside of HIV infection. The research indicates that there may be several shared, underlying pathophysiological mechanisms of biological significance that have clinical as well as treatment implications for the comorbidity. The clinical reality is that—regardless of the potential diagnoses of other depressive disorders and their severity—the specific comorbidity of MDD and HAND is relatively common, of particular clinical concern in its own right, and remains unstudied.

Regarding one pathophysiological mechanism, MDD has been associated with pro-inflammatory cytokine secretion. In addition, several antidepressants have been observed to mediate anti-inflammatory changes in these same cytokines. Nevertheless, a relationship between improvement in inflammatory biomarkers and the remission of MDD has not been consistently observed. It should be noted, however, that these studies largely relied upon measurements of inflammation from peripheral blood rather than CNS-derived measures. This is an important consideration because chronic neuroinflammation is the most common form of brain injury in treated PWH. Indeed, the absence of effective, combination antiretroviral therapy (going forward referred to simply as “ART”) has been linked to higher rates of depressive disorders. Further, among PWH receiving ART, a significant relationship has been observed between depression and elevated inflammatory biomarkers in both the peripheral blood and the cerebrospinal fluid (CSF). Detection of CSF HIV RNA has also been clearly correlated with depressive disorders. These findings suggest that MDD and HAND do not simply co-occur but rather overlap in their pathophysiologic mechanisms. This hypothesis would explain the continued high prevalence rate of MDD along with recurrent episodes among PWH, that some forms of MDD (manifesting more with apathy than depressed mood) in PWH are treatment-resistant, and that there is an increased likelihood that MDD will increase the risk for cognitive decline among PWH. Thus, therapies that target both inflammatory mechanisms and mechanisms of virologic persistence may be successful in treating both conditions simultaneously.

Given the evidence of lowered CSF and brain tissue DA levels in the natural history of HIV–associated neurocognitive disorder, DA agonists have been recommended for study. Supportive preliminary data were previously reported from case studies of carbidopa and L-DOPA. Pramipexole is a clinically effective non-ergot DA agonist approved for Parkinson’s disease with full intrinsic activity and high selectivity for interacting with the D2 sub-family of DA receptors (associated with its Parkinson’s disease efficacy). It also has a preferential activity for D3 receptors (associated with its reported antidepressant efficacy). The D3 receptor activity of pramipexole may be specifically responsible for a positive response in MDD, particularly for the subset of symptoms often seen among PWH – i.e., apathy, lethargy, and social withdrawal. Animal and human studies indicate a pattern of relationship between DA deficiencies, such as those seen in PWH, with chronic neuroinflammation, that includes pro-inflammatory cytokine-induced reductions in the synthesis and release of DA.

Another potential mechanism related to inflammation can be viewed through the window of the gut microbiome and the microbiome-gut-brain (MGB) axis. One of the hallmarks of HIV disease is a rapid and profound depletion of CD4 cells in the gut-associated lymphoid tissue, resulting in an enteropathy with increased translocation of microbial products, despite ART. The microbiome–gut–brain axis is a bi-directional communication system between the CNS and gut microbiota. It plays a role in maintaining neuroendocrine and neuroimmune homeostasis as well and impacts several neurologic and neuropsychiatric disorders, including MDD and HAND Intestinal bacteria are known to modulate the host metabolome, and circulating metabolites can enter the CNS directly, affecting CNS activation or interacting with the immune system – thus, indirectly affecting brain function. Furthermore, short chain fatty acids – as products of colonic bacterial fermentation – can drive microglial maturation, which has been implicated in the development of neurodegenerative diseases, along with the neurodegenerative aspects of HAND, and in depression, In fact, depression has been referred to as a microglial disease (Yirmiya et al., 2015). Additional data are required to target the most appropriate treatment of the comorbidity of MDD with HAND as well as to better define the pathophysiological underpinnings of this comorbidity in terms of neuroinflammatory, neuroendocrine, virologic, and microbiomic mechanisms.

Moving onward to the specific foci of this article, since the introduction of effective, combination ART for HIV infection, depressive disorders (particularly MDD) and HAND have emerged as the most prominent neuropsychiatric disorders affecting PWH in the United States. Estimates of HAND range from 25 to 47% of the population of PWH with access to ART, depending upon the definition of HAND, the screening tests used, the comorbidity burden, the populations sampled, and prevalence of confounding conditions (Cysique et al., 2004; Heaton et al., 2010; Heaton et al., 2011; Sacktor et al., 2016). Depressive disorders generally have been reported in as few as 18 to as many as 81% of PWH over the entire era of ART (Bing et al., 2001; Ciesla and Roberts, 2001; Gaynes et al., 2008; Simoni et al., 2011; Arseniou et al., 2014; Eller et al., 2014; Nanni et al., 2015). Both disorders result in substantial morbidity and early mortality in PWH; yet, their relationship to one another, remains to be established, by category of HAND (Antinori et al., 2007) and by systemic HIV disease stage and treatment history historically (Centers for Disease Control [CDC], 1992). Antidepressant therapies, particularly stimulating antidepressants, have also been used in the treatment of HAND. Thus, identifying therapies specific for the comorbidity, optimizing the treatment of depressive disorders and HAND, while minimizing toxicity, is urgently needed. In this article, we review the differentiation of depressive symptoms from MDD along with the status of “depression” and MDD specifically in the current era of effective ART in which plasma viral load is routinely suppressed to non-detectable levels in PWH, while discussing sub-groups most impacted by stigmatization and associated life stressors. In addition, we differentiate cognitive symptoms from HAND and its sub-types. Further, we propose an organizing theoretical model for a pathophysiological substrate of the comorbidity of MDD with HAND determined by ongoing inflammation despite suppressive ART. We also delineate sources of inflammation across sites, including the CNS, the microbiome, and the vasculature, and relate them to the proposed model. Moreover, we specifically integrate the inflammatory process with DA depletion in terms of a dual relationship with MDD and HAND in PWH. Finally, the therapeutic implications of this theoretical model from the comorbidity are discussed with respect to a preference for anti-inflammatory agents. and agents that increase DA transmission.

## “Depression” – A matter of definition

MDD is more common among PWH than the general population (Bing et al., 2001; Ciesla and Roberts, 2001; Gaynes et al., 2008; Simoni et al., 2011; Arseniou et al., 2014; Eller et al., 2014; Nanni et al., 2015). It is the second most common cause of years lost to disability in the United States overall (Global Burden of Disease Study 2013 Collaborators, 2015). Furthermore, among PWH, depressive disorders have been suggested to contribute to decreased antiretroviral (ARV) medication adherence and poor clinical outcomes – nationally and internationally (Kacanek et al., 2010; Sheth et al., 2015; Uthman et al., 2014). In contrast to HAND, where there remains no consensus-driven approach to treatment – apart from effective. combination ART itself with sustained viral suppression and treatment of comorbidities affecting cognitive dysfunction – MDD has long been treated with antidepressants having established efficacy and safety in PWH (Ferrando and Freyberg, 2008). Yet, MDD in this population remains generally under-diagnosed and under-treated (Asch et al., 2003). There are no data to date on treatment of the comorbidity of depressive disorders with HAND in PWH. Thus, both identifying new therapies for HAND and optimizing the use of existing therapies for depressive disorders in PWH are urgently needed in order to address the treatment of the comorbidity.

Related to the minimal investigation of the comorbidity to date, we will focus upon how depressive disorders interact with HAND in the current era of ART. First and most importantly, “depression” is a highly non-specific term that does not refer to any one clinical entity or group of entities. The most common use of the term “depressed” is to describe an individual’s level of depressed or sad mood and associated changes– i.e., level of depressive symptoms. This definition of “depression” is useful in that it can describe anyone in the general population in terms of a level of depressed mood that one might experience and that most people do experience at times in their everyday lives. However, the level of depressive symptoms may only apply to a person at the moment of their self-report or for a brief specific time period prior to their self-report, as captured by numerous psychometric instruments. The “level of depressed symptoms” at any particular time does not necessarily correspond to the likelihood of a presence of psychopathology with depressive features, i.e., a “depressive spectrum disorder.” The latter – “depressive spectrum disorders” –represents another definition of “depression,” though embracing numerous related entities. These may be identified – only in part – on the basis of level of depressed mood. They are also identified based upon the presence versus absence of specific constellations of associated symptoms and, sometimes, also by the circumstances under which the depressed mood and associated symptoms occur. While the assessment for risk for depressive disorder has certainly been promulgated by questionnaires alone, the use of a measure of depressive symptom level in the general population cannot properly be used to define the presence of an actual mental health disorder diagnosis by “cut-off scores” or other similar score-based projections or algorithms – as these disorders represent qualitatively different expressions of “depression” that impair daily life functioning.

The depressive disorder most commonly recognized by primary care providers is MDD. However, MDD under-represents the heterogeneity of depressive disorders. “Syndromal depression” can take a number of forms. One of the most common is actually “adjustment disorder with depressed mood,” which occurs with reference to a stressful life event after which there is a maladaptive response with deficits in functional status directly related to the impact of a life stressor. Another form is “persistent depressive disorder” defined in the recent text revision of the DSM-5 (American Psychiatric Association [APA], 2022) as a consolidation of the prior DSM-IV diagnoses of “chronic major depressive disorder” and what had been referred to as “dysthymic disorder.” Further complicating matters, persistent depressive disorder can occur concomitantly with an episode of MDD. Moreover, there are “substance-induced depressive disorders,” “medication-induced depressive disorders,” and “depressive disorders due to another medical condition.” Finally, personality disorders can present with prominent depressive symptoms (e.g., borderline personality disorder). Thus, it may be concluded that “depression” – from the syndromal point of view – represents an entire spectrum of disorders, all of which can deleteriously impact the functional status of PWH.

One specific situational context in which depressive symptoms have been recognized to occur is with respect to the progression of HIV disease clinically (Atkinson et al., 2008). As treatment has evolved today, it is highly unusual for PWH to be vulnerable to clinical symptoms due to HIV. When PWH are significantly non-adherent to ART, they might experience a symptom referable to HIV and may no longer be able to continue to maintain the frequent denial that HIV will ever cause any illness. If such a non-adherent PWH fails to mount a positive, resilient coping response to an HIV-specific symptom that occurs, the subsequent, day-to-day uncertainty about the future may disposes the person to higher levels of depressive symptoms and depressive disorders. In the current era of ART, this is a very uncommon scenario, particularly in high-resource countries, despite the high frequency of depressive disorders.

Yet, PWH are still confronted with a high life stressor burden (including multiple, intersecting stigma–related life experiences, including homophobia, and racism) that pose a risk for depressive disorders more frequently than in the general population (Tran et al., 2019). In addition, they are more likely to have limited social support and maladaptive coping experiences, which limits their ability to generate a resilient response to life stressors (Riaz et al., 2020). Moreover, they have an increased likelihood to have history of MDD prior to the diagnosis of HIV infection, which predisposes them to subsequent episodes. These facts are generally under–appreciated, as is the high prevalence of depressive disorders among PWH today. In addition, PWH typically represent demographic groups from the general population that are already at high risk for depressive disorders premorbidly because of health inequities, poverty, psychoactive substance use, and experiences of marginalization. Side effects of medications prescribed to PWH, including certain ARVs [efavirenz and dolutegravir particularly] have been associated with depression. Medical comorbidities observed at increased frequency among PWH, such as HCV co-infection, CVA and MI are also associated with depression. Moreover, with ART guidelines having recommended universal treatment since 2012 (United States Department of Health and Human Services [USDHHS], 2012) and with the WHO having embraced those recommendations in 2015 (World Health Organization [WHO], 2015), PWH today survive over a much longer period of time than in the past – allowing for a greater period of time for these predisposing factors to be evidenced as depressive disorders.

The most common reason for the wide prevalence range of depressive disorders across studies of PWH is the lack of focus upon a clear-cut definition of the type of depression “phenotype” or “biotype” defined by the methods employed. Very few studies specifically focus on defining a phenotype or biotype of “depression.” For example, depressive disorder categories are commonly defined using the manual-guided, Structured Clinical Interview for the DSM (“SCID”) (First et al., 2015), but not an underlying phenotype or biotype. Of all of the depressive disorder categories, MDD is the most important in terms of the literature documenting associated medical consequences of excess morbidity and early mortality. Other reasons for the variability in “depression” prevalence across studies include differences in samples studied, techniques employed for enrolling participants, and the assessment tools used during the study. In addition, the complex interplay between psychosocial and biomedical factors seen with MDD among PWH can render the valid diagnosis of comorbid depressive spectrum disorders highly challenging (Gaynes et al., 2008; Simoni et al., 2011; Eller et al., 2014; Uthman et al., 2014). Yet, making the diagnosis is critically important, as one meta-analysis showed that treatment for depression improves the odds of ART adherence by 83% (Sin and Dimatteo, 2014).

## Depression in the current era of ART

For the aforementioned reasons, we will focus here on the most well-studied of the depressive spectrum disorders amongst PWH – MDD. The mechanisms mediating the high prevalence of MDD in the setting of HIV infection are multi-factorial and may be associated with neuroendocrine, immunologic, and inflammatory changes (Del Guerra et al., 2013; Felger and Lotrich, 2013). A cascade of life stressors related to the impact of a recent HIV diagnosis may exacerbate physiological changes induced by HIV infection, which may jointly contribute to the greater propensity for MDD observed in this patient population. Of note, it appears that ART itself might lower the risk for MDD – compared to ART-naïve patients, in a study defining clinically significant depression by adverse events reported as depression requiring drug therapy and as suicide attempts (Gutiérrez et al., 2014). This effect may occur through decreased HIV-induced immunologic activation and inflammatory responses occurring with ART. Despite the well-known and dramatic reductions in morbidity and mortality that have occurred since the introduction of effective, combination ART, depressive disorders continue to occur commonly among PWH – with current predictions that they will remain one of the leading causes of disability in the future in the general population (Mathers and Loncar, 2006). This context underscores the importance of diagnosing and treating depressive disorders to maximize the benefits of ART.

Recent reports link depression to mortality among PWH, though not entirely consistently. One study failed to show a significant relationship between ever-reported depressive symptom [by a Patient Health Questionnaire (PHQ)-9 score > 10] and mortality in a large sample of PWH (Bengtson et al., 2017). In contrast, another study – of women with HIV [in the Women’s Interagency HIV Study (WIHS) from 1998−2011] showed that among 848 women contributing 6,721 cumulative years of follow-up, the greatest mortality occurred in women who had reported depressive symptoms and had not initiated ART. The hazard ratio for depressive symptoms was 3.38 [95% confidence interval (CI): 2.15, 5.33] and for ART was 0.47 [95% CI: 0.31, 0.70] (Todd et al., 2017). A third study by Gustafson et al. (2017) also used data from this same WIHS study. However, their analyses used the Veterans Aging Cohort Study (VACS) Index (as an HIV biological index predictor); another constructed index (the Fried Frailty Index); and a third “mental health index” [depressed mood level using the Center for Epidemiological Studies Depression scale (CES-D)] as the mortality predictors. Yet, both the VACS index and the frailty index are actually multi-variable indices themselves, whereas depressed mood level was the only predictor that truly represented a single measure and a single construct. Hence, while the outcome was that depressed mood level was not found to have independent predictive power on mortality against that of the two constructed indices, it was the only variable tested alone. Moreover, overlapping variance in mortality that would have been ordinarily assigned to the depressed mood variable could have been assigned to the multi-variable indices there instead – despite the attempt of the authors to modify the frailty measure to partly account for overlapping depressive symptoms with the frailty construct. Further, the variance in mortality accounted for by depression could have been truncated by their use of depressed mood level rather than syndromal depression as the depression predictor. A more recent study (Pence et al., 2018) comprised an observational clinical cohort of 5,927 patients with two or more assessments of depressive severity who were receiving HIV primary care at six academic medical centers in the United States from 2005 to 2015. Consecutive depressive severity measures were converted into a time-updated measure: percentage of days with depression (PDD), following an established method for determining depression-free days. Compared with patients with no follow-up time with depression (PDD, 0%), those with the entire follow-up time period with depression (PDD, 100%) demonstrated a 37% increased risk of missing appointments, a 23% increased risk of a detectable plasma viral load, and a doubled mortality rate. Given the fact that over 60% of PWH in the United States today are over 50 years old, the interest in the relationship of depression to mortality is yet greater for older PWH. Of note in this regard, a more recent study (Turrini et al., 2020), in turn, sampled Medicare beneficiaries aged 65 and over from 2011 to 2016, including 100% of those having HIV infection (*N* = 43,708) as well as a random 1% of the sub-sample of persons without HIV (*N* = 1,029,518). A Cox proportional hazards model assessing mortality showed that older Medicare beneficiaries with HIV showed 3.6 times the hazard for mortality versus older persons without HIV. There are two paths by which “depression” might lead to an increased risk for mortality: (1) depression could decrease ART adherence thereby increasing HIV replication, and increasing clinical morbidity causing mortality and/or (2) depression could increase deleterious neuroendocrine responses (including hypercortisolemia) leading directly to immunocompromise (including a decreased Th1:Th2 cytokine ratio), and increasing clinical morbidity leading to early mortality. Regardless, the linkage of depression with mortality remains the overriding result. Of note, Cook et al. (2004) reported that depressed participants receiving mental healthcare were significantly less likely to die from AIDS-related causes than were depressed participants not receiving mental healthcare. While a relationship between depressive symptoms and poor HIV disease outcomes might have been expected there, it is noteworthy that the relationship between depressive symptoms and mortality in the study were documented independently of ART use and adherence.

On a separate note, MDD is a potentially lethal disease in its own right, as well as in association with primary medical comorbidities other than HIV infection. It is the most prevalent mental health disorder and the psychiatric diagnosis most commonly associated with suicide (Henriksson et al., 1993; Harwood et al., 2001). Studies suggest that nearly the majority of those who die by suicide suffer from affective disorders and that the majority of people who complete suicide are depressed at the time of their deaths, with MDD increasing the risk of a suicide attempt sevenfold in the 2 years after discharge from a hospital (Bostwick and Pankratz, 2000; Brown et al., 2000; Orquendo et al., 2006). Thus, MDD with imminent risk for suicide is a serious, potentially lethal condition that requires immediate intervention. Moreover, epidemiological studies of MDD have consistently demonstrated higher prevalence amongst women (Cyranowski et al., 2000). This occurs for reasons that we do not entirely understand. Among women with HIV, it may be of particular importance that they have increased adverse childhood experiences (frequently referred to as “ACEs”) associated with trauma and post-traumatic stress disorder, including sexual abuse prior to puberty and the death in childhood of a family member (not due to suicide or homicide) that might mediate poor ART adherence later in life (Cuca et al., 2019). Future research should examine the interventions that are not only the most effective but also the least toxic for women with HIV having a comorbidity of MDD. The results of the research on mortality with MDD in PWH rise to a level of importance strongly arguing for the prioritization of integrated clinical care of PWH. This would optimally include pro-active mental healthcare monitoring for the occurrence of depressive disorders and a linkage with entry into care providing effective treatment. This approach should also be integrated with monitoring for decreased ART adherence and sub-clinical as well as clinical signs of increased morbidity due to HIV.

## HAND in the current era of ART

There are three levels of severity represented by HAND: (1) asymptomatic neurocognitive impairment (ANI), (2) mild neurocognitive disorder (MND), and (3) HIV-associated dementia (HAD) (Antinori et al., 2007). ANI is a condition that occurs when there is impairment in two or more domains of neurocognitive performance assessed by standardized testing without any concomitant decline in functional status in activities of daily living. In actuality, it represents a “condition” rather than a “disorder” in that it is defined by a lack of impairment in functional status. It has been generally reported to be the most common of the three categories of HAND in research studies in the current era of ART. MND, is characterized by mild neurocognitive impairment (NCI) in at least two domains of neurocognitive performance along with mild impairment in functional status. As there is a decrement in functional status, it represents a true “disorder” and is the second most common of the HAND categories. In HAD, there is at least moderate NCI present in two or more domains of neurocognitive performance accompanied by at least moderate impairment in functional status that – like for the other categories of HAND — is specifically due to HIV-associated NCI (rather than due to unrelated or residual deficits in functional status that might be sequelae of other HIV-associated illnesses). The neurocognitive disorders of HIV infection recapitulate systemic HIV disease progression represented by CDC staging (Centers for Disease Control [CDC], 1992), though the latter is infrequently useful to characterize patients today. The earliest stage of HIV disease progression is asymptomatic HIV infection (CDC stage A) (analogous to no NCI or to ANI), followed by early symptomatic HIV infection (CDC stage B) (analogous to MND), followed, in turn, by the late stage/AIDS (CDC stage C) (analogous to HAD). For each HAND category, there should be no evidence of another pre-existing cause of NCI, including systemic and CNS opportunistic infections, non–HIV–associated neurological diseases (including neurodegenerative diseases and cerebrovascular disease), NCI associated with history of coronary artery disease, traumatic brain injury (TBI), CNS toxicities due to prescribed medications, or any other potential confounding general medical or psychiatric disorders (e.g., MDD and alcohol or substance use disorders).

## Sex assigned at birth, gender role, age, MDD, and HAND

Aside from psychosocial factors, a number of demographic and biological factors have been associated with MDD among PWH. Sex has long been recognized as a risk factor for MDD in the general population, with women generally showing twice the risk of men (Hasin et al., 2018). The same appears to be the case for women with HIV (Morrison et al., 2002; Simbayia et al., 2007), though not without exception (Rubin et al., 2019). This depression risk, in turn, may be related to greater mortality risk for women with HIV (Cook et al., 2004; Todd et al., 2017). Women with HIV and depression have been reported to have five times the likelihood of impaired executive control cognitively versus women without HIV who are depressed and three times the likelihood of impaired executive control versus men with HIV and depression (Rubin et al., 2019). It has also been reported that women with HIV more frequently show NCI independently of depression, which has been supported on multivariate analysis (Robertson et al., 2014). However, this difference remains to be fully established, as the results have not been consistent by cognitive domain, and studies conducted thus far have had insufficient statistical power to examine this effect (Burlacu et al., 2018; Maki et al., 2018). In addition, sex differences observed today might be related to a lower reading level among women with HIV (Sundermann et al., 2018). While it has been reported that the difference persists with control of educational status, this finding was related to performance on a brief screening test (the International HIV Dementia Scale) rather than on a neurocognitive battery (Duarte et al., 2021).

Although it might well be expected that lifetime depression would increase with age, this has not always been the case in the literature. Research examining the issue indicates that the pattern of lifetime prevalence observed in cross-sectional epidemiologic studies might best be explained by MDD incidence declining with age, together with some respondents failing to recall past MDD episodes (Patten et al., 2010). With regard to PWH, since 2018 over 50% of PWH in the United States have been 50 years of age or older, and by 2030 it is anticipated that 70% will be over age 50 (Wing, 2016) and 25% of PWH will be over age 65. Rates of depression amongst older PWH are similar – not lower – than those of younger PWH (De Francesco et al., 2019). Of special relevance here, the occurrence of MDD is associated with internalized HIV stigma, likely to be yet higher among older women (Vyavaharkar et al., 2010).

Regarding gender issues, gender micro-aggressions by cis-gender people are as likely to be met with aggressive responses as gender non-conforming behavior is among transgender people. Non-conforming, gender-related abuse over time has been documented to lead to increased levels of depression. Stigma and oppression have been reported to have an additive effect impacting those stigmatized over their lives – effectively explained using the minority stress theory. Meyer (2003) defined the minority stress concept combining impacts of factors, including issues such as poverty, racism, sexism, heterosexism, and cissexism, as having an additive effect on health, mental health and related patient care outcomes. Earnshaw et al. (2013) noted that the National HIV/AIDS Strategy reported on the vulnerability of disenfranchised populations, including transgender people, to infection with HIV as well as the role of stigma as a risk factor increasing HIV infection risk. It appears to that the intersection of sexism, cissexism, and racism in the form of stigma-related trauma exacerbates the risk of HIV infection as well as higher levels of loneliness. Further, there is an increasing severity of depression in people with multiple minority identities. This relates to transgender women of color being the highest risk group for MDD. It might also be expected that older women with HIV with undiagnosed and untreated MDD would be lonely, stigmatized, and have multiple minority identities, which would place them at particularly high risk for ART non-adherence and detectable plasma viral loads.

## The interaction of depression and HAND: a possible link to inflammation

Depressive disorders may contribute to or confound the diagnosis of HAND and – for this reason – MDD specifically was listed as an exclusion criterion for HAND in the Frascati conference criteria that re-defined these diagnoses in 2007 (Antinori et al., 2007). It should be noted that the exclusion of any major depressive episode was applied to ANI, while the exclusion of a severe major depressive episode was applied to MND and HAD. As ANI is the most common HAND condition in the current era of ART, the implications of any MDD diagnosis regardless of its severity are a significant concern. MDD does have a direct overlap with HAND – e.g., decreased ability to think or concentrate or indecisiveness (Antinori et al., 2007; American Psychiatric Association [APA], 2022), which is a criterion for the diagnosis of MDD in the DSM-5-TR [for which one of us (KG) served on the review group for neurocognitive disorders]. Perhaps, the more specific issue should not be the severity of MDD *per se* but the specific occurrence of neurocognitive dysfunction as a symptom of a major depressive episode. The relationship of the syndrome of MDD to neurocognitive performance is different than that of depressive symptoms generally. In addition, it should be added that there are numerous depressive disorders other than MDD which can also manifest with disturbances of neurocognitive function – including persistent depressive disorder (formerly dysthymia), depressive episodes due to bipolar affective disorder, disruptive mood dysregulation disorder, premenstrual dysphoric disorder, adjustment disorder with depressed mood, substance–induced depressive disorder, depressive disorder due to cerebrovascular disease, and depressive disorders due to a general medical condition. HAND and the spectrum of depressive disorders are the most common neuropsychiatric disorders among PWH. Hence, to exclude one from being comorbid with the other, even when severe, is not in keeping with the clinical realities of the day-to-day patient care of this patient population. While it might be considered that a diagnosis of HAND could be deferred pending the effect of antidepressant therapy, it must also be noted that cognitive symptoms may persist, not infrequently, after other depressive symptoms have resolved.

Of special note in this context, PWH with an incident major depressive episode in the CNS HIV Antiretroviral Therapy Effects Research (CHARTER) study have been shown to have a greater prior likelihood of having a higher CSF viral load – in contrast to plasma viral load (Hammond et al., 2016). At study entry there, 18% of participants overall were found to have a detectable CSF viral load – despite a non-detectable plasma viral load. Over 18 months of follow-up, the cumulative incidence of elevated CSF viral load compared to plasma load was significantly higher amongst those with MDD compared to non-depressed participants. Also, MDD has proven to be a significant discriminator of detectable CSF viral load against not only plasma viral load but also CNS penetration effectiveness of ARVs, duration of ART, ART adherence, and race (Hammond et al., 2014).

Another study (Cysique et al., 2016) evaluated 95 PWH who were clinically stable on ART. They found that lifetime and recent MDD history were more strongly associated with decreased independence in daily living and increased neurocognitive complaints than with baseline neurocognitive performance. Yet, lack of remission, instability on treatment for MDD over time, and severity of symptoms in current MDD were relevant for baseline neurocognitive performance. It was concluded that recurrent episodes of MDD, the chronicity profile of MDD, and associated comorbidities were keys factors relevant to neurocognitive performance among PWH. Hence, not only do depressive disorders and HAND occur together but also there may be a recursive relationship between these groups of disorders over time.

## Inflammation in HAND

Although current ART regimens suppress HIV replication to non-detectable levels in the CSF and in the peripheral blood, the inflammatory process continues – though reduced – in the face of a non-detectable plasma viral load (Eden et al., 2007). In some persons with a non-detectable plasma viral load, HIV RNA is detectable in the CSF. This occurs in approximately 10% of individuals who undergo lumbar puncture in clinical practice (Eden et al., 2010; Rawson et al., 2012). Impaired neurocognitive performance has been associated with detection of CSF HIV RNA, even when assays with a sensitivity as low as 1 to 2 copies/ml CSF have been used (Anderson et al., 2021). Nevertheless, the magnitude of CSF load has not closely correlated with neurocognitive deficits in the current era of ART. This is more than likely due in part to the fact that viral load does not reflect either viral protein expression or the related inflammatory response to viral proteins. A recent study shows that there is ongoing, significant transcription without whole virion production and that these transcripts drive the inflammatory response and are associated with brain tissue injury (Suzuki et al., 2022).

The inflammatory process (and possibly primary immune dysregulation) unrelated to viral replication (or protein expression) may well be the primary neuropathophysiological mechanism of HAND. Several inflammatory measures are relevant regarding the progression of HAND. These include MCP-1, sCD14, SDF-1alpha and TNF-alpha, all of which were associated with neurocognitive worsening in the CHARTER study (Marcotte et al., 2013). Neopterin, reflecting macrophage activation, is a longstanding neuroinflammatory biomarker that retains associations with NCI in PWH in the current era of ART. Other inflammatory biomarkers of interest for NCI in PWH have been identified, including sCD163, IL-6, IL-1, IFN-γ, and IL-8 (Williams et al., 2021). CXCL10, a 10-kDa IFN-γ-inducible protein (formerly referred to as IP-10) belonging to the CXC chemokine family, is secreted in response to IFN-γ from monocytes, endothelial cells, fibroblasts, astrocytes, and lymphocytes; elevated levels of CXCL10 have been correlated with severity of NCI in PWH (Mehla et al., 2012). GlycA, a composite blood biomarker of glycosylated proteins reflecting acute phase reactants (including alpha1-acid glycoprotein and haptoglobin) indicating inflammation in studies of coronary artery disease has recently been related to impaired neurocognitive performance among PWH (Ances et al., 2021). In addition, the neurodegenerative marker of neuro-axonal injury, neurofilament light chain (NFL), has been associated with NCI in PWH by numerous studies and may reflect outcomes resulting from specific pro-inflammatory profiles over the course of the disease and its treatment (Gisslen et al., 2021). It might be the case that chronic immune activation in relation to viral persistence and – amongst older PWH – immunosenescence will prove to be of greater pathophysiological import than primary virologic mechanisms in HAND, though their relative contribution by age against that of increased viral transcription remains to be established.

**FIGURE 1:**
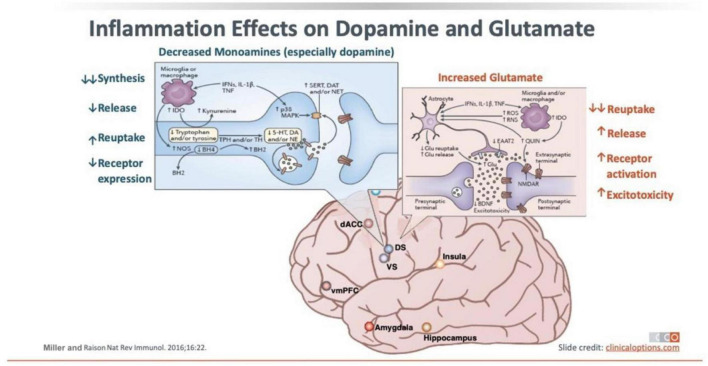
Inflammation effects on dopamine and glutamate.

Regarding the association of neuroinflammation with depression, there is significant evidence demonstrating that elevated levels of selected cytokines in the periphery (IL-1, IL-6, and IFNγ) and other pro-inflammatory mediators (high sensitivity C-reactive protein or hsCRP) influence the occurrence and progression of MDD episodes (Kim et al., 2016; Al-Hakeim et al., 2018) as well as decreased DA signaling and increased glutamate release with associated excitotoxicity (see Figure 1). Inflammation in the CNS, induced by the presence of HIV, either (Miller and Raison, 2016) actively replicating or in its integrated state, confers activation of microglia, which, in turn, promotes a pro-inflammatory M1 macrophage milieu that triggers astrogliosis, neuronal dendritic shortening, and decreased DA signaling. Collectively, this inflammatory cascade can induce the symptoms of MDD. As well, gene expression profiling on post-mortem brain tissue from antidepressant medication-free individuals with a history of MDD has shown dysregulation of an entire profile of pro- and anti-inflammatory cytokines (Enache et al., 2019). Moreover, stressful life events (including the stigma associated with HIV and PWH) are associated with the onset of major depressive episodes in the promotion of the inflammatory cascade, while antidepressant therapy has been observed to mediate anti-inflammatory changes associated with increases in IL-10 (Roque et al., 2009; Laumet et al., 2018; Carboni et al., 2019). In addition, antidepressant therapy has been associated with increases in BDNF and hippocampal neurogenesis (Zhang et al., 2016), and, related to comorbid HAND, the hippocampus is known to be preferentially affected by HIV with a high proviral load. Yet, a relationship between improvement in inflammatory biomarkers and resolution of depression has not always been observed, although it should be noted that these studies largely relied upon measurements of inflammation in the peripheral blood.

Among PWH, the absence of ART is clearly linked to higher rates of depressive disorders (Gutiérrez et al., 2014). As has been noted, in individuals receiving ART, a significant relationship between depressive disorders and inflammatory biomarkers in both the peripheral blood and the CSF has been reported. Likewise, the detection of CSF HIV RNA has also been correlated with MDD risk. Downstream effects of increased pro-inflammatory cytokine secretion with decreased intracellular levels of free glutathione and increased oxidized glutathione, malondialdehyde (MDA) levels, and isoprostane levels would be expected (Buckley et al., 2021). One study has reported increased reactive oxidative species (ROS) levels and lower levels of intracellular glutathione, in macrophages from PWH compared to healthy participants (Morris et al., 2012). Another more recent study revealed that PWH with MDD had a significantly dysregulated oxidant/anti-oxidant balance, though the intricacies of the relationships observed there need to be further delineated (Rivera-Rivera et al., 2014). These additional data help to elucidate that the issue of neuroinflammation is at least as powerful – and likely greater – among PWH with MDD than it is among persons with MDD in the general population. In fact, neuroinflammation might be further exacerbated when comorbid HAND is present as well, despite variations in the extent of CNS tissue damage related to historical differences between dates of contraction of HIV infection and ART initiation amongst PWH. However, we are not aware of any studies to date that examine the specific sub-group of PWH with comorbid HAND and MDD, among whom we might expect that a true synergism could be present between those two disorders with regard to the extent of neuroinflammatory responses that they induce. Future research in this area could provide important insights into the role of inflammation in these conditions. While it can be seen that there is significant overlap between the pro-inflammatory changes demonstrated with HAND in PWH and those demonstrated to occur with MDD, the interaction of neuroinflammatory changes with regard to the comorbidity of HAND and MDD (or other depressive disorders) remains unknown.

## Ethnoracial life stressors, intersecting stigmas, inflammation, and neurocognitive impairment among PWH

An increasing number of neuroinflammatory biomarkers associated with NCI have been identified related to ethnoracial factors among older PWH (Kompella et al., 2021). Given the additive effect of ethnoracial disparities manifesting through social determinants of health together with a high allostatic load due to a traumatic life stressor burden most likely to impact vulnerable PWH in line with the “minority stress model,” the sub-group of PWH with multiple ethnoracial identities demonstrates higher levels of inflammatory biomarkers, immunologic deficits and clinical sequelae (Flentje et al., 2020). Thus, this sub-group is also at high risk for NCI and depressive disorders along with the functional status decrements associated with them. It has been reported that the additive impact of a chronic reaction to traumatic life stressors, particularly those occurring in childhood, is particularly impactful among women with HIV (Tedaldi et al., 2015; Watson et al., 2019). Poor ART adherence by PWH is a further factor potentiating an increased level of inflammation impacting neurocognitive function. Additionally, HIV infection itself depletes DA in brain through Tat (Miller et al., 2018) and also depletes 5-HT levels (Shah et al., 2019) – compounding the number of factors likely to increase the risk for depressive disorders as well as neurocognitive disorders associated with HIV.

## The interaction of HAND and depression in PWH: a possible link to the microbiome-gut–brain (MGB) axis

The MGB axis is a bidirectional communication system between the CNS and the enteric microbiome that aids in maintaining neuroimmune homeostasis (Osadchiy et al., 2019). Alterations in the gut microbiome are known to impact a number of neurological and neuropsychiatric disorders, including MDD and possibly HAND, though studies on the latter are limited (Sharon et al., 2016; Fung et al., 2017; Osadchiy et al., 2019). Communication between the enteric microbiome and the CNS occurs directly and indirectly through the action of microbial metabolites, including short chain fatty acids (SCFAs), tryptophan metabolites, neurotransmitters, and secondary bile acids, which can translocate into the circulation (Sharon et al., 2016; Fung et al., 2017; Osadchiy et al., 2019). Microbial metabolites directly interact with colonic epithelial cells, enteroendocrine cells, and the mucosal immune system, as well as indirectly communicate with the CNS via afferent vagal stimulation (Osadchiy et al., 2019). Enteric bacteria produce neurotransmitters and induce colonic cells to produce neurotransmitters, such as DA, 5-HT, GABA, norepinephrine, and histamine – all of which can be active in the CNS, directly affecting neuronal activity as well as interacting with the immune system (Yano et al., 2015; Mittal et al., 2017; Chen et al., 2021; Huang and Wu, 2021). Indeed, the majority of 5-HT is derived from the gastrointestinal tract, where microbial metabolites promote colonic enterochromaffin cells to produce 5-HT (Yano et al., 2015).

Tryptophan can be synthesized and catabolized by enteric bacteria. Low levels of circulating tryptophan have been found in PWH – despite adequate dietary tryptophan intake (Murray, 2003; Cervenka et al., 2017; Wang and Qi, 2019). The majority of tryptophan is not anabolized to 5-HT but, in fact, is catabolized through an alternative pathway – the kynurenine pathway. Among PWH, the rate of tryptophan catabolism through the kynurenine pathway is increased and associated with clinical disease progression (Wang and Qi, 2019). Indoleamine-2,3-dioxygenase 1 (IDO-1), one of two enzymes responsible for converting tryptophan to kynurenine, is induced during HIV infection and itself induces inflammation – in part related to the downstream generation of quinolinic acid (Peirce and Alviña, 2019). IDO-1 activity is associated with disrupted mucosal immunity and microbial translocation and is only partially reduced by ART (Taleb, 2019; Wang and Qi, 2019). Bacterial community taxonomic distributions correlate with the catabolism of tryptophan and elevated *T*-cell activation, including specifically members of the *Enterobactereiaceae* and *Staphylococcaceae* family, which are enriched with the progression of HIV infection. Further, bacteria with homologues to tryptophan catabolic enzymes are enriched in enteric disease-associated microbiota among PWH, including *Pseudomonas*, *Xanthomonas*, *Burkholderia*, *Stenotrophomonas*, *Shewanella*, and *Bacillus* (Vujkovic-Cvijin et al., 2013). Enteric microbial communities associated with the progression of HIV infection potentially lead to increased enteric epithelial destruction and pathogen overgrowth. The kynurenine catabolism pathway can also deplete the pool of tryptophan available for anabolism to 5-HT in PWH, increasing the risk for MDD.

While there is less evidence for DA interacting with the MGB axis in MDD than for 5-HT, emerging data also support the involvement of MGB signaling in DA release, synthesis, and bioavailability (Hamamah et al., 2022). Relevant microbial genera – *Prevotella, Bacteroides*, *Lactobacillus*, *Bifidobacterium*, *Clostridium*, *Enterococcus*, and *Ruminococcus* – intricately involved with DA pathways via effects on DA precursors, enzymes, receptors, transporters, and metabolites have been investigated. States of intestinal dysbiosis involving these genera have been demonstrated to disrupt MGB signaling, leading to DA deficits that manifest in neuropathological conditions, such as Parkinson’s disease (Hill-Burns et al., 2017; Liu et al., 2017; Franco et al., 2021). Overall, the literature supports the possibility that Parkinson’s disease begins within the gastrointestinal tract. It might be noted that Parkinsonism was associated with HIV infection historically before the advent of effective, combination ART. Therefore, enteric modulation of both DA and 5-HT may have indirect effects on the brain related to the response to treatment of both MDD and HAND with antidepressants among PWH.

Neuroinflammatory markers have been identified that are associated with both MDD and HAND. The persistence of HIV within the CNS and the associated resulting neuroinflammation appears to drive the clinical expression of HAND over time. This process includes an interaction with the MGB axis. Specifically in HIV infection, sCD14 and sCD163 – markers of monocyte activation – have been reported to correlate with peripheral trafficking of activated, HIV infected myeloid cells into the CNS as well as with CNS inflammation and activation referable to resident cells. The amount of virus in trafficking myeloid cells may be much lower than that in trafficking *T*-cells. One of the hallmarks of HIV infection is a rapid and profound depletion of CD4+ *T*-cells in the gut-associated lymphoid tissue (GALT) (Brenchley et al., 2006), resulting in an enteropathy (Brenchley, 2013) with an increased translocation of microbial products, including inflammatory bacterial endotoxin, despite ART. As aforementioned, the MGB axis is known to impact a number of neurological and neuropsychiatric disorders, including MDD (Sharon et al., 2016; Fung et al., 2017; Kang et al., 2017; Khan and Oloketuyi, 2017), and the limited studies examining the gut microbiome in HAND suggest an alteration in the gut microbiome in those with HAND as well (Zhang et al., 2018; Dong et al., 2021). Microbial (or microbial metabolite) translocation from the gut has been implicated in the process of CNS immune activation and HIV infection with associated, subsequent neurocognitive dysfunction. In addition, SCFA products of colonic bacterial fermentation drive microglial maturation (Caspani et al., 2019), which is also of special interest related to HAND neuropathogenesis – as microglia are directly infected by HIV and as their maturation relates to cellular function.

Prior work has shown that *Ruminococcus bromii*, a keystone species in the production of anti-inflammatory SCFAs, is depleted among PWH (Monaco et al., 2016). A pilot study of probiotic supplementation in PWH showed neurocognitive improvement and decreased CSF neopterin levels occurring with probiotic supplementation (Ceccarelli et al., 2017), suggesting that the gut microbiome plays a role in the pathophysiology of HAND and, possibly, in its response to treatment. Though less is known specifically about the role of the MGB axis in MDD among PWH, a role of the MGB axis has been implicated in MDD in the general population (Caspani et al., 2019; Peirce and Alviña, 2019). Alterations in the gut microbiome in MDD compared to healthy controls have been observed; however, the bacterial taxonomic results between studies have not been entirely consistent (Caspani et al., 2019), indicating that functional analysis of bacterial pathways may be necessary to further detail the association between the MGB axis and MDD, generally, and with MDD among PWH, specifically.

## The interaction of HAND and depression: the vascular factor

With the advent of effective, combination ART, increased attention was paid to a vascular factor having significant relevance for HAND. Regarding the CNS, early evidence indicated that the blood-brain barrier (BBB) was maintained over the course of HIV infection. Subsequent research suggested that the BBB was, in fact, “leaky” (Persidsky et al., 2016). Contributing factors included pro-inflammatory cytokines secretion, HIV Tat protein, and cocaine use as well as metabolic changes that occur with long-term use of effective, combination ART. Systemically, it was noted early on that long-term exposure to the protease inhibitors (with associated insulin resistance and diabetes risk) and abacavir particularly (amongst the nucleoside reverse transcriptase inhibitors) is associated with an increased risk for coronary artery disease and myocardial infarction (Laurence et al., 2018). Of greater relevance to current ART regimens, recent research has suggested that a toxicity of the integrates inhibitors can contribute to both coronary artery and cerebrovascular disease (Neesgaard et al., 2022). This effect seems to be limited to the first 2 years of therapy with the greatest effect at treatment onset followed by a linear decrease over time. Support for a vascular effect of HIV itself (generally considered inflammatory) is derived from studies using multiple methodologies, including transcranial Doppler studies (TCD), carotid intima-media thickness (IMT) studies, and studies of the frequency of internal carotid arterial plaques (Grima et al., 2011). The implications of these vascular effects have been borne out by studies of clinical outcomes – transient ischemic attack (TIA) and cerebrovascular accident (CVA) (Janjua et al., 2017). Thus, vascular changes have been documented to contribute to the pathogenesis of HAND. It has also been well described that MDD is associated with vascular compromise and disruption of frontal-subcortical and limbic networks involved in mood regulation. This has been documented specifically in late life MDD (Jellinger, 2021). Thus, the convergence of these vascular factors might be of particular importance in the comorbidity of MDD with HAND, perhaps yet more so among older PWH. While it is not possible to fully address these vascular factors within the scope of this article, the reader is referred for additional information to: Aizenstein et al. (2016) and to: Cysique and Brew (2019).

## The interaction of depression and HAND: a neurotransmitter linkage with dopamine and inflammation

CSF levels of homovanillic acid (HVA), the major terminal metabolite of DA, are consistently lower in people with depression that in the general population, as evidenced by a recent meta-analysis (Ogawa and Kunugi, 2019). This has been supported in the setting of HIV infection in a study of 123 PWH and 102 persons without HIV (Saloner et al., 2020). Lower CSF DA levels complement the foregoing findings reported with HVA (Berger et al., 1994). CSF studies are needed, though, intriguingly, it has been suggested that peripheral DA levels may reflect central DA activity (Mackie et al., 2018). In other research focused on DA transmission, it was shown that lower CSF levels correlated with higher levels of depressive symptoms (Lian et al., 2020). Obermann et al. (2009) observed that the substantia nigra of PWH was hyperechogenic by transcranial ultrasound relative to controls and that this hyperechogenicity was correlated with decreased DA levels in the CSF, decreased CD4 cell counts, and impaired motivation and performance on psychomotor tests. The results indicated that changes in the nigrostriatal system in PWH precede prominent extrapyramidal symptoms and neurocognitive dysfunction. These studies are relevant not only for PWH but also for treatment of MDD in the general population, among whom decreased DA transmission also occurs with MDD. Moreover, studies overwhelmingly support decreased DA concentrations following chronic HIV viral protein exposure. gp120 has been shown to reduce DA uptake in mesencephalic neuronal cultures *in vitro*. In addition, the intra-striatal injection of gp120 leads to a specific loss of DA. Tat also has established neurotoxicity for DA neurons due to excitotoxicity and mitochondrial dysfunction (Ferris et al., 2008). NCI, including attention deficits, as well as apathy associated with HIV infection, further support the clinical relevance of DA deficits among PWH (McLaurin et al., 2021). Moreover, there are currently no data supporting that ART restores these DA deficits, in terms of improvement of the phenylalanine (Phe) to tyrosine (Tyr) ratio, which is increased prior to ART – reflecting decreased conversion of phenylalanine to tyrosine, a rection catalyzed by phenylalanine hydroxylase and the rate-limiting enzyme in the biosynthesis of DA (Zangerle et al., 2010). Further, microglial infection specifically might serve as the pathophysiological source of chronic DA deficits among PWH. As aforementioned, in the era prior to effective, combination ART, brain involvement in HIV infection would present with Parkinsonian motor symptoms. Some time ago case studies aimed at DA replacement using carbidopa and L-DOPA in neurocognitive disorder among PWH, as with Parkinson’s disease, and supported the premise of the clinical relevance of reversing the DA deficit (Kieburtz et al., 1991). The psychostimulants, particularly methylphenidate, have been used as a pathogenesis-directed treatment for NCI among PWH (Fernandez et al., 1988; van Dyck et al., 1997). Those results suggested that effective treatment of MDD as well as neurocognitive disorder among PWH might best be treated with DA agonists, such as pramipexole, or other agents that increase DA transmission, such as bupropion and venlafaxine.

Identification of therapies that surpass the limitations of current ART regimens and optimize the treatment of both MDD and HAND, particularly with respect to improving DA transmission, remain urgently needed today. Animal and human studies indicate a relationship between chronic inflammation and deficiencies in DA transmission (Felger and Miller, 2012). Of note, related to the prior discussion of a neuroinflammatory linkage having emerged as a key contributor to the pathogenesis of MDD and HAND in PWH, pro-inflammatory cytokine-induced reductions in the synthesis and release of DA have been demonstrated. Moreover, DA receptors (D2 and D3 receptors) on microglia and astrocytes in the CNS are specifically known to modulate the neuroinflammatory response.

## Neuroimaging, inflammation, dopaminergic transmission, and a link with treatment

Neuroimaging techniques using TSPO PET for neuroinflammation reflecting microglial activation may further support a neuroinflammatory basis for this comorbidity. PWH show global increases in TSPO expression in brain compared to controls (Vera et al., 2016). Increases in TSPO expression were associated with NCI (Vera et al., 2016). In addition, TSPO PET studies have shown increased TSPO expression with MDD, correlated with its severity (Setiawan et al., 2015). Some evidence indicates decreased levels of TSPO expression in response to antidepressant treatment (Richards et al., 2018).

Yet, several issues have emerged from these early studies. For example, the first-generation tracer, PK–11195, was not highly specific for TSPO and has a low signal-to-noise ratio. In addition, the second-generation tracer, PBR28, requires genotyping for potential TSPO polymorphisms prior to its use (Owen et al., 2012). While third-generation tracers are now available, such as GE–180, data are very limited. Moreover, questions have been raised concerning whether TSPO is the best ligand for neuroinflammation in brain. TSPO has been related to astrocytic as well as to microglial activation. To address this issue, new tracers for the membrane purinergic receptor P2X7 (P2X7R) are in development (Zhang et al., 2021). However, the differentiation of astrocytic versus microglial activation remains to be conclusively demonstrated for this ligand as well.

Molecular-targeted neuroimaging has become well-established for evaluating abnormalities in DA transmission. ^123^I-ioflupane SPECT can be used to quantify DA transporter (DAT) binding potential in striatal DA neurons of the caudate nucleus and putamen. Decreased striatal DA transporter (DAT) expression has been identified in MDD both post-mortem and *in vivo* (Pizzagalli et al., 2019). In addition, PET scanning has been used to quantify D2/D3 receptor occupancy in both striatal and extra-striatal brain regions (Leung, 2018). Patients with depression showed greater D2/3 receptor availability in several striatal regions, including the bilateral ventral pallidum/nucleus accumbens and the right ventral caudate and putamen. Currently, neuroimaging techniques are not sufficient to reliably differentiate D2 from D3 receptor occupancy, which is of interest, as D3 receptor occupancy has been more directly related to anhedonia. It can be concluded that neuroimaging techniques can make a major contribution to identifying both the neuroinflammatory and the specific neuroendocrine mechanisms manifesting clinically with comorbid MDD and HAND – suggesting that they may also prove to have significant clinical utility both for establishing diagnoses and monitoring treatment response of these disorders in the future.

Taken together, these findings suggest that neuroinflammation is a pathophysiologic mechanism affecting DA pathways in the pathogenesis of MDD and HAND among PWH. Several categories of antidepressants including selective serotonin reuptake inhibitors (SSRIs)/serotonin reuptake inhibitors (SRIs) mediate anti-inflammatory changes. However, a relationship between inflammatory biomarkers and the resolution of MDD with antidepressant therapy has not been consistently observed, though IL-10 has been noted to be a regulatory cytokine decreasing inflammation and associated with antidepressant treatment response (Roque et al., 2009; Laumet et al., 2018; Carboni et al., 2019). Nevertheless, the search for the development of innovative, new therapeutic drugs continues for anti-inflammatory agents specifically effective in the CNS for the treatment of MDD, HAND and now their comorbidity among PWH.

Therapies that specifically target DA pathways associated with neuroinflammation may have the potential to treat MDD, as well as – possibly yet more effectively–comorbid MDD and HAND in PWH. Pramipexole is a safe and clinically effective non-ergot DA agonist and FDA-approved medication for Parkinson’s disease with full intrinsic activity and high selectivity for the D2 family of DA receptors. Previous studies of pramipexole have also established safety and efficacy for Parkinsonian symptoms/Parkinsonism and for restless legs syndrome (Gossard et al., 2021). While the efficacy of pramipexole in PD has been mapped to agonism at the D2 receptor, its efficacy for MDD has been mapped more closely to the D3 receptor, though to the D2 receptor as well (Tokunaga et al., 2012). For anhedonia in MDD, pramipexole binding to D3R in extra-striatal brain regions was associated with treatment efficacy of MDD (Ishibashi et al., 2011). In addition, DA deficiencies have been reported in comorbid HAND (McLaurin et al., 2021). Thus, we anticipate pramipexole, and more recently FDA-approved DA agonists, to be likely to prove specifically effective for the treatment of the comorbidity of MDD with HAND among PWH.

## The interaction of depression and HAND: treatment implications

The foregoing findings suggest that depressive disorders (particularly MDD) and HAND may share a common pathophysiologic mechanism and that therapies targeting CNS inflammation could potentially be successful in treating both conditions. The first line of treatment for comorbid depression and HAND that might be considered is effective, combination ART itself. One study of 602 patients (302 not on ART and 300 on ART) followed for the first 12 months of HIV care in Uganda (Wagner et al., 2012) reported a greater decrease in depression amongst ART-treated than non-ART-treated patients in an intention-to-treat, multivariate analysis. When change in physical health functioning was added to the regression models, ART treatment status still predicted positive longitudinal change on measures of depression and hopelessness. Thus, ART treatment status remained a significant independent predictor of mental health outcomes, with most mental health benefits of ART occurring over the first 6 months of care. These data suggest that ART may be an effective treatment for depressive disorders, supporting the relevance of inflammatory processes in depressive disorders generally and the possibility for a preference of CNS-penetrating antiretroviral medications particularly.

With regard to other potential treatments for comorbid depressive disorders and HAND, the antidepressants, as a group, will now be considered. Coleman et al. (2012) have reported on an integrative care approach using measurement-based care for depressive disorders with PWH in a 124-patient clinical chart review. They reported significant reductions in depression and plasma viral load as well as significant increases in CD4 cell count with antidepressant treatment. It was concluded that this approach to care appears to be an effective method of improving depression, virologic and immunologic outcomes among PWH. Of note, for those PWH who have been previously treated with antidepressants for prior major depressive episodes, it might be optimal to re-institute antidepressant treatment as maintenance care upon the initiation of ART – regardless of the absence of a current major depressive episode. Such antidepressant treatment would be considered a standard of care for prophylaxis of recurrence of depressive episodes in MDD long-term. As a result, there would also likely be a decreased likelihood of subsequent ART regimen changes due to blips in plasma viral load induced by non-adherence. For a similar reason, antidepressant treatment might best be considered to be the primary treatment versus psychotherapy in the case of the occurrence of a first major depressive episode over the course of HIV infection in patients already treated with effective, combination ART. The potential for differential efficacy of specific antidepressant treatment modalities merits consideration in this patient population.

The psychostimulants have been used widely as a treatment for HIV-associated NCI since the early stages of the epidemic and are also known to be effective agents in treatment-resistant MDD (known as “TRD”). These medications are well known to increase DA transmission. Psychostimulants increase norepinephrine release in the prefrontal cortex, which alters the firing pattern of DA neurons resulting in changes in action potential-dependent DA release, in turn, affecting the temporal pattern of DA release in the nucleus accumbens. Regarding the effects of psychostimulants on cognition, significant improvement was found in one trial prior to effective, combination ART in which early symptomatic and AIDS patients (*n* = 20) were treated with methylphenidate 10 mg po tid (to a maximum of 90 mg/day) over 2 weeks – with a cross-over to dextro-amphetamine 15 mg po bid (to a maximum of 60 mg/day), if there was no response (Fernandez et al., 1988). Another study examined the efficacy of sustained-release methylphenidate in a small sample of PWH having NCI treated with methadone in a double-blind placebo-controlled crossover trial. They found improvement from baseline on a composite neurocognitive performance measure with methylphenidate but not with placebo, though performance on drug did not differ significantly from placebo performance (van Dyck et al., 1997). More recently, modafinil was studied in a placebo-controlled trial in which PWH were selected for clinically significant fatigue and were evaluated on neurocognitive performance based upon ten neurocognitive tests (McElhiney et al., 2009). Improvement was greater in the modafinil group than in the placebo group, though this effect was not observed in a study of armodafinil (Rabkin et al., 2011). These few studies aside, there remains insufficient research on the long-term use of the psychostimulants in this setting and the potential for abuse must be considered when employing these agents.

Given the evidence of lowered CSF and brain tissue DA levels in the natural history of HAND, DA agonists have been recommended for study. Further, supportive preliminary data exist from case studies of carbidopa and L-DOPA as DA precursors in HAND (Kieburtz et al., 1991). As previously noted, the DA agonist, pramipexole, has preferential activity for D3 receptors (associated with antidepressant efficacy). Its D2 receptor affinity could relate to motor effects that might treat HAND patients with associated Parkinsonism – though rare today – and possibly to more general deficits involving the motor domain – such as psychomotor slowing – known to occur frequently among PWH. The D3 receptor activity of pramipexole may be specifically responsible for a response of a subset of symptoms associated with depressive disorders that are also seen in HAND – i.e., apathy, lethargy, and social withdrawal – whereas the cognitive depressive symptoms (helplessness, hopelessness and low self-worth) and the typical affective symptoms (crying spells) are more typically absent with MDD among PWH. Like the psychostimulants, pramipexole has established clinical trial data to support its use in TRD with an efficacy of 40–74% (Hobson et al., 1999). Pramipexole is generally well-tolerated with manageable side effects of sedation, dizziness and nausea as well as orthostasis. However, pramipexole does carry the infrequent toxicities of sleep attacks, impulsive behavior (including pathological gambling) and visual hallucinations (occurring in about 2%). Another DA agonist, ropinirole, has shown efficacy in TRD of 40–44% (Hobson et al., 1999). While ropinirole has a superior safety profile with respect to the foregoing infrequent toxicities of pramipexole, the side effects of sedation, dizziness and nausea are yet more common with ropinirole. Moreover, recently ropinirole was studied as an adjunctive treatment for depression, and no difference in outcome was detected between the ropinirole adjunct treatment group and the no-adjunct control group (Gershon et al., 2019). It was noted that these results were counter to expectation and that this may relate to a difference in pharmacological activity profile between ropinirole and pramipexole – favoring pramipexole.

**FIGURE 2:**
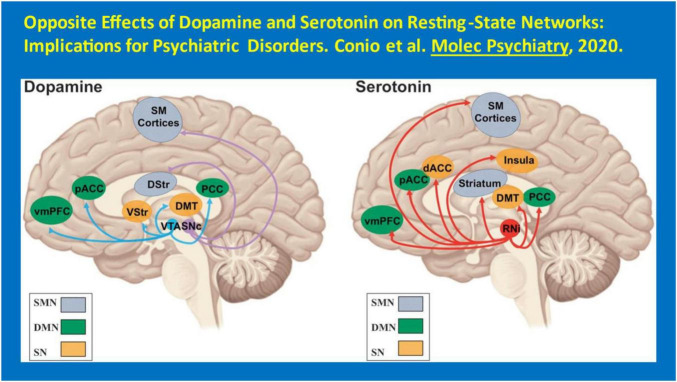
Opposite effects of dopamine and serotonin on resting-state networks: implications for psychiatric disorders. Conio et al. (2020) Molec Psychiatry.

Another neurotransmitter noteworthy in this context of the need for additional treatments for HAND is 5-HT. 5-HT has been shown to have low levels in the CSF of PWH (Kumar et al., 2001). Thus, SSRIs and SRIs, more generally, may play a role in the pathogenesis of MDD as well as HAND among PWH. SSRIs as a group appear to diminish apoptosis, a mechanism of neuronal cell death in HIV infection, and have also been used to decrease inflammation surrounding an infarct acutely after a CVA. Moreover, antidepressants increasing serotonergic transmission are known to be effective boosters of host immunity in HIV/AIDS (Orlander et al., 2009). Further, these medications appear to foster suppression of plasma viral load (Letendre et al., 2007; Tsai et al., 2010). Citalopram, sertraline and trazodone may improve neurocognitive performance (Letendre et al., 2007), though this remains to be convincingly demonstrated. It must be stated that there remains limited prospective evidence yet accumulated to demonstrate that the SSRIs or SRIs actually are therapeutically useful in the context of HAND, with the exception of one small trial of paroxetine (Sacktor et al., 2018) (see Figure 2). Of note, DA signaling is associated with alterations in resting-state networks, specifically an increase in functional connectivity and activity in the sensorimotor network and in the salience network as well as a concurrent decrease in functional connectivity and activity in the default–mode network. This pattern is associated with psychomotor activation and salience of sensory stimuli consistent with the alleviation of MDD) (Conio et al., 2020). 5-HT signaling is associated with a decrease in sensorimotor network activity along with an increase in default–mode network activity. This pattern is associated with psychomotor inhibition and a predominance of internal thought consistent with increased severity of MDD. Inflammatory markers such as C-reactive protein (CRP) and ESR (erythrocyte sedimentation rate) have been noted to be significantly elevated with MDD, and SSRIs (such as escitalopram and fluoxetine) have reduced them – independent of their antidepressant effects (Chavda et al., 2011). SSRIs have also been suggested to affect both hippocampal plasticity and neurogenesis in both animal models and clinic patients. Chronic escitalopram treatment restores spatial learning, monoamine levels, and hippocampal long-term potentiation in an animal model of depression (Bhagya et al., 2011). Fluoxetine actually decreases memory retention in rats pre-treated with ghrelin (Carlini et al., 2007). Thus, there may be differences in the neurocognitive impact amongst the SSRIs. Preliminary evidence suggests that – unlike their effects on MDD– specific SSRI/SRI agents may be differentially useful to treat HAND. This area merits additional research attention.

Of further relevance regarding comorbid MDD and HAND is the pathophysiology of MDD and the historic work referred to as the “serotonin hypothesis,” dating back to 1969 that proposed the central import of disturbances of tryptophan metabolism (Lapin and Oxenkrug, 1969). Nearly 95% of tryptophan is metabolized *via* the kynurenine pathway (Muneer, 2020). There is a shunt of dietary tryptophan pool utilization from 5-HT production to kynurenine production. The greater the activity of this shunt, the greater is the risk for MDD. In addition, it has also been shown that decreased pyridoxine activity relates to decreased serotonin anabolism as well, as it is a co-factor in the decarboxylation of 5-hydroxytryptophan to 5–HT. This has been specifically demonstrated by our group in the setting of bereavement amongst PWH (Baldewicz et al., 1998). However, there are two steps of the tryptophan-kynurenine pathway: (a) formation of kynurenine from tryptophan and (b) post-kynurenine metabolism. We have discussed the relevance for the treatment of MDD of the former. The post-kynurenine pathway is also of interest here. Prior to it is the cleavage of the indole ring of tryptophan, which results in the formation of N-formylkynurenine followed by kynurenine (Oxenkrug, 2010). The rate-limiting enzyme of kynurenine formation from tryptophan is indoleamine 2,3-dioxygenase (IDO) in astrocytes, microglia, and microvascular endothelial cells. As noted, this pathway produces the post-kynurenine metabolite and NMDA receptor agonist, quinolinic acid, which is associated with neuroinflammation and neurotoxicity. IDO activity appears to be higher in the CNS of PWH, given that the CSF tryptophan/kynurenine ratios are significantly lower among them, compared to persons without HIV. Moreover, this pathway yields free radical generators, 3-hydroxykynurenine and 3-hydroxyanthranilic acid (Mackay et al., 2006). Increased formation of NMDA receptor agonists is expected to result in a hyper-glutamatergic state now recognized to be consistent with MDD and known to be associated with HAND as well.

Of relevance, experimental and clinical data both demonstrate that TNF-alpha and IFN-gamma can trigger MDD via stimulation of IDO and a consequent increase of kynurenines formed from tryptophan (O’Connor et al., 2009a). It is possible that genetic and environmental (e.g., frequent major stressful life events, inadequate social support and maladaptive coping) factors increase both the risk for and severity of MDD and HAND by upregulation of tryptophan-kynurenine catabolism. Of further note here, the effects of genetic factors (such as high producer alleles of pro-inflammatory genes) are mediated by cytokine-induced up-regulation of IDO. High producer alleles of TNF-alpha genes result in high production of these cytokines and can trigger the “super-induction” of IDO (Oxenkrug, 2010). Moreover, pro-inflammatory cytokine–induced stimulation of cortisol production and augmentation of activation of IDO by stressor hormones suggest that the tryptophan-kynurenine pathway might be a synergistic point of gene-environmental interaction relevant to comorbid MDD and HAND. Therapeutically, these data suggest that TNF–alpha antagonists such as etanercept and infliximab as well as cortisol antagonists such as DHEA might be helpful in the treatment of MDD, HAND, and their comorbidity.

Converging evidence suggests that kynurenine activity in the blood parallels its activity in the brain. For example, increased plasma IDO activity is coupled with simultaneous increases of kynurenine and quinolinic acid levels in the CSF subsequent to IFN-α treatment (Baranyi et al., 2015; Dantzer, 2017). Further, pre-clinical data have specifically linked peripheral and central IDO activation to MDD-like behaviors (Raison et al., 2009), while peripheral inhibition of IDO blocked the central transcription of IDO in the brain and the development of MDD-associated symptoms following immunological stimulation (O’Connor et al., 2009b). This work is supported by the recent report of increased CSF quinolinic acid levels along with concomitant increased IL-6 levels in suicidal individuals compared to healthy controls (Erhardt et al., 2013), and the kynurenine pathway has been specifically associated with suicidality. Despite ART, CSF quinolinic acid levels were significantly higher in PWH compared to persons without HIV and were associated with higher phosphorylated Tau levels, which, in turn, correlated with decreased prospective memory (Anderson et al., 2018). Thus, a clinical trial of IDO inhibitors might be indicated for comorbid MDD and HAND, such as 1-methyl tryptophan.

Beyond DA and 5-HT, glutamate is a widespread and abundant (6.0−12.0 mM) neurotransmitter that is associated with excitotoxic neuronal cell death, one of the primary mechanisms of neuronal cell death associated with HAND (Haughey et al., 2001). N-acetyl aspartate (NAA) is a brain tissue metabolite measure used in MR spectroscopy (MRS) that is related to this mechanism – since NAA binds glutamate in neuronal cells (as N-acetyl-aspartyl glutamate). With a drop in NAA, this binding capacity decreases, and more glutamate is available to induce excitotoxicity. Regarding PWH, MRS studies have focused on the combination of glutamate and glutamine, as the “Glx” peak. Glutamate and glutamine are highly related in brain, as glutamate is thought to be taken up by astrocytes, converted to glutamine, transported back to the pre-synaptic neuron, and converted back to glutamate (Rothman et al., 1999) – posited as the “glutamate/glutamine shuttle” hypothesis (Kimmich et al., 2002; Shank and Campbell, 1984). Hence, one would expect that an NMDA receptor antagonist, such as memantine, would be effective in the treatment of HAND. Yet, memantine failed to show a therapeutic effect in ACTG 301, focused on the treatment of HIV-associated dementia (Schifitto et al., 2007). Perhaps, an effect might have been more likely to be observed in the earlier stages of HAND in which less neuronal cell death had occurred. With respect to MDD, this same mechanism has also been focused upon for treatment. While approved and widely used for the induction and maintenance of anesthesia via IM or IV administration, ketamine, as well as esketamine (S-ketamine, the S enantiomer of ketamine) are NMDA receptor antagonists of special interest for MDD. The mechanism of action of esketamine is distinct from conventional monoaminergic antidepressant treatments and esketamine profoundly affects fast excitatory glutamate transmission – increasing brain-derived neurotrophic factor release and stimulating synaptogenesis. Results to date suggest that these drugs might treat MDD – and suicidality specifically –more rapidly than the currently FDA-approved antidepressants (Kowalczyk et al., 2021). They may also be specifically effective in the treatment of comorbid HAND and MDD. In contrast, data to date related to acetylcholine and decreased cholinergic transmission are minimal with respect to HAND –unlike Alzheimer’s disease.

## Conclusion

MDD and HAND are the most prevalent neuropsychiatric disorders that imapct PWH, resulting in significant morbidity and early mortality. While virologic factors remain of relevance and vascular factors play a role, increasing evidence derived from neurochemical/neurotransmitter, neuroimmunological, neuropathological, and neuroimaging studies suggests that neuroinflammation may represent a common pathophysiological linkage driving a potential synergy between these two disorders. Specifically, a linkage has been demonstrated between neuroinflammation and decreased DA transmission. Decreased DA transmission has been associated, in turn, with both MDD and HAND. Our proposed empirically based theoretical model regarding the comorbidity of MDD and HAND links differing sources of evidence demonstrating that neuroinflammation and DA transmission deficits are independently involved in both conditions and might well determine associations with clinical status and therapeutic outcomes to a yet greater extent in their comorbidity. To date, limited research has been done to study a pathophysiologic relationship between MDD and HAND, and no formal research has been conducted on the treatment of these two disorders through a pathophysiologic linkage. Given the anticipated significant frequency of the comorbidity of MDD and HAND and its potentially greater impact than either disorder taken alone, a call to further study of this area is indicated. Of special interest is the evidence that MDD in PWH might be more specifically mediated by DA transmission than is the case with MDD in the general population. This indicates the potential utility of therapeutic agents predominantly aimed at improving DA transmission, such as DA agonists. Yet, most currently prescribed antidepressants are based on impact on other neurotransmitters—5-HT, norepinephrine, and glutamate. Ultimately, a common modality of treatment for both MDD and HAND could simplify therapy, reduce toxicity, and increase efficacy in this patient population.

## Data availability statement

The original contributions presented in this study are included in the article/supplementary material, further inquiries can be directed to the corresponding author.

## Author contributions

All authors listed have made a substantial, direct, and intellectual contribution to the work, and approved it for publication.
